# Molecular Characterization of Dermatophytes Isolated in a Tertiary Care Hospital in the North Eastern Part of India

**DOI:** 10.7759/cureus.74815

**Published:** 2024-11-30

**Authors:** Chakraborty P, Valarie Lyngdoh, Neeta Gogoi, Sony Gandhi, Denzel L Lyngdoh, Sheryl Lanong, Kunjjal Shah

**Affiliations:** 1 Microbiology, North Eastern Indira Gandhi Regional Institute of Health and Medical Sciences, Shillong, IND; 2 Clinical Microbiology, North Eastern Indira Gandhi Regional Institute of Health and Medical Sciences, Shillong, IND; 3 Biotechnology, North Eastern Indira Gandhi Regional Institute of Health and Medical Sciences, Shillong, IND

**Keywords:** blastn, dermatophyte, fasta, internal transcribed spacer( its), sequencing

## Abstract

Introduction

Dermatophytes are the most common cause of superficial fungal infection. They are mostly diagnosed using phenotypic methods, but recently the molecular methods seem to be gaining ground. The objective of the present study was to compare the phenotypic and genotypic methods of identification of dermatophytes and understand the feasibility of using molecular methods for routine diagnosis of dermatophytosis.

Methods

Eight repertoire isolates of *Trichophyton *species identified using conventional phenotypic methods were sequenced using a target of the internal transcribed spacer (ITS1 & ITS2) flanking the DNA sequence 5.8S rDNA. The FASTA sequences obtained were put under the Basic Local Alignment Search Tool - Nucleotide (BLASTn) in the National Center for Biotechnology Information (NCBI) blast portal and compared with existing sequences.

Results

The comparison between the phenotypic and molecular methods was established in 7/8 (87.5%) of the isolates. The ITS-based sequencing was able to speciate *Trichophyton rubrum* but failed to differentiate between the species complexes of *Trichophyton mentagrophytes *and* T. rubrum.*

Conclusion

ITS-based molecular identification is unable to correctly compare phenotypic and molecular methods of identification. Moreover, due to the high polymorphism inherent in dermatophyte species complexes, ITS was unable to delineate the dermatophyte species correctly.

## Introduction

Fungal diseases account for the fourth most prevalent disease globally [1], and among them, superficial fungal infection is the most common, with a worldwide prevalence of 20-25% [2,3]. Superficial fungal infection has progressed from being an innocuous disease to now becoming a disease of public health concern, especially with an alarming increase in the number of difficult-to-treat cases. The main causative agents are the keratinophilic dermatophytes, namely, *Trichophyton* sp., *Epidermophyton* sp., and *Microsporum* sp., leading to the disease condition known as dermatophytosis or tinea. The prevalence has been calculated throughout India and falls within a very wide range of 6.09%-61.5% [4]. Until 1935, the predominant species reported worldwide was *Trichophyton mentagrophytes;* the trends changed towards *Trichophyton rubrum* and remained as such until recently when *T. mentagrophytes *again gained ground. Studies have the limelight on features of anthropization in *T. mentagrophytes *where a propensity of acclimatization to human host has been observed contributing to the easy transmissibility and the sudden thrust in cases [5].

Dermatophytosis has always remained a domain of the dermatologist with much reliance on clinical presentation and examination under a Wood’s lamp. In recent years, an increase in the number of difficult-to-treat cases has caused most clinicians to seek the help of laboratories. Laboratory diagnosis of dermatophytosis has heavily relied on conventional mycological procedures of direct microscopy with 10-40% potassium hydroxide (KOH) mount and culture on solid media such as Sabouraud dextrose agar (SDA), SDA with chloramphenicol and cycloheximide (SCCA), and dermatophyte test medium (DTM). However, these culture-based techniques are surrounded by their own limitations; the culture yield, even in direct mount positive samples, does not exceed 60-75% [6]. Molecular techniques are gaining ground, and identification of dermatophytes is being achieved either by using species-specific primers or pan-fungal gene targets [7,8]; amongst them, the most commonly used is the polymorphism of the internal transcribed spacer (ITS1 & ITS2) flanking the DNA sequence 5.8S rDNA [5].

The present study aims to analyze the phenotypic and genotypic modalities of identification in a subset of dermatophytes isolated in the department of microbiology in a tertiary care hospital in the north eastern part of India. It also intends to analyze the feasibility of using molecular methods as the cornerstone of laboratory diagnosis with respect to dermatophytosis in comparison to conventional methods.

## Materials and methods

Diagnosis of dermatophytosis was done at the Department of Microbiology at North Eastern Indira Gandhi Regional Institute of Health and Medical Sciences, Shillong, Meghalaya, a tertiary care teaching hospital in the north eastern part of India, after duly obtaining exemption of ethical clearance from the Institute Ethics Committee. The phenotypic identification of dermatophytes was based on direct visualization of septate fungal hyphae in 10%-40% KOH solution followed by culture in SDA, SCCA, and DTM and incubation at 25°C and 37°CC for a period of four weeks. Subsequent to morphological identification, colonies of dermatophytes were maintained by regular subcultures on SDA slants. The present study was conducted on eight such phenotypically identified dermatophyte isolates from repertoire stocks.

The extraction of genomic DNA from the dermatophytes was done as per the manufacturer’s guidelines using the QIAamp® DNA Mini Kit. The fungal mycelia were suspended in phosphate-buffered saline and centrifuged for 10 mins at 7,500 rpm, followed by resuspension in 180 mL of 2 mM ethylenediaminetetraacetic acid (EDTA) solution and incubation for 30 min at 37°C. Then, 20 µL of proteinase K and 200 mL of lysis buffer were added and vortexed with incubation of this mixture at 56°C for 30 min and then 95°C for 15 min to deactivate the proteinase K. Additionally, 200 mL of lysis buffer was added, followed by transfer of this mixture to a bead tube with further vortexing for 10 mins at maximum speed and incubation at 90°C for two hours. Subsequently, 200 mL of chilled ethanol at 4°C was added, followed by transfer of the mixture to a QIAamp Mini spin column. The mixture was washed with 500 mL of wash buffer with centrifugation each time at 8,000 rpm for one minute, and 200 mL of elution buffer was added, followed by centrifugation at 8,000 rpm for one minute to get the final elute of dermatophytic DNA. The DNA was checked in a nanodrop and stored at -20°C for further downstream analysis [9].

Molecular identification was performed by nucleotide sequence analysis of ITS of 5.8SrDNA with the same primers as that of amplification. The amplicons obtained were sent for Sanger sequencing to Rajiv Gandhi Centre for Biotechnology (RGCB), Thiruvananthapuram, India.

Following sequencing, the obtained sequences were then subjected to Basic Local Alignment Search Tool- Nucleotide (BLASTn) analysis to determine their identity. BLASTn, accessed via the NCBI (National Center for Biotechnology Information) BLAST online platform (https://blast.ncbi.nlm.nih.gov/) was used for general-purpose nucleotide sequence alignment tasks and to detect alignments between somewhat divergent sequences [10].

## Results

In total, the present study was conducted with the right number of repertoire fungal culture isolates. These isolates were phenotypically identified as *T. rubrum* (three isolates), *T. mentagrophytes* complex (four isolates), *Trichophyton violaceum* (one isolate).

Following the sequencing and analysis process, eight sequenced samples yielded clean, high-quality sequences ready for BLASTn analysis. Reverse sequence samples were processed using the Reverse Complement platform to obtain their respective forward sequences.

The BLASTn analysis yielded significant matches to established sequences within the NCBI nucleotide database. Sample 1 displayed a 99.77% identity match with the sequence identified by Subject ID OR244359.1, corresponding to the organism *Trichophyton indotineae*; other notable matches include a 99.77% identity with sequence OR088161.1, which is associated with the organism *Trichophyton interdigitale*, and a 99.77% identity with sequence OR018228.1, which is linked to *T. mentagrophytes*. A complete list of significant matches is available in Table 1.

**Table 1:** BLAST analysis results for query DNA sequences BLASTn: Basic Local Alignment Search Tool - Nucleotide

Query	Subject ID	Organism	Description of mRNA	Max Score	Total Score	Query Cover	Percent identity	Acc. Length
Sample 1	OR244359.1	Trichophyton indotineae	Small subunit ribosomal RNA, internal transcribed spacer 1, 5.8S ribosomal RNA, internal transcribed spacer 2, and large subunit ribosomal RNA	815	815	100%	99.77	676
	OR088161.1	Trichophyton interdigitale	Internal transcribed spacer 1, 5.8S ribosomal RNA, internal transcribed spacer 2, and large subunit ribosomal RNA	815	815	100%	99.77	756
	OR018228.1	Trichophyton mentagrophytes	Internal transcribed spacer 1, 5.8S ribosomal RNA, and internal transcribed spacer 2	815	815	100%	99.77	544
Sample 2	OR244359.1	Trichophyton indotineae	Small subunit ribosomal RNA, internal transcribed spacer 1, 5.8S ribosomal RNA, internal transcribed spacer 2, and large subunit ribosomal RNA	913	913	100%	99.8	676
	OQ978648.1	Trichophyton interdigitale	internal transcribed spacer 1, 5.8S ribosomal RNA, internal transcribed spacer 2, and large subunit ribosomal RNA	913	913	100%	99.8	686
	OQ787386.1	Trichophyton mentagrophytes	Internal transcribed spacer 1, 5.8S ribosomal RNA, internal transcribed spacer 2, and large subunit ribosomal RNA	913	913	100%	99.8	648
Sample 3	OR374017.1	Trichophyton rubrum	Internal transcribed spacer 1, 5.8S ribosomal RNA, and internal transcribed spacer 2	606	606	100%	100	560
Sample 4	OR374017.1	Trichophyton rubrum	Internal transcribed spacer 1, 5.8S ribosomal RNA, and internal transcribed spacer 2	494	494	70%	96.63	560
	OR206381.1	Trichophyton soudanense	Internal transcribed spacer 1, 5.8S ribosomal RNA, and internal transcribed spacer 2	494	494	70%	96.63	471
	OP434592.1	Trichophyton kuryangei	Internal transcribed spacer 1, 5.8S ribosomal RNA, and internal transcribed spacer 2	494	494	70%	96.63	644
Sample 5	OR244359.1	Trichophyton indotineae	Small subunit ribosomal RNA, internal transcribed spacer 1, 5.8S ribosomal RNA, internal transcribed spacer 2, and large subunit ribosomal RNA	773	773	100%	97.77	676
	OR088161.1	Trichophyton interdigitale	Internal transcribed spacer 1, 5.8S ribosomal RNA, internal transcribed spacer 2, and large subunit ribosomal RNA	773	773	100%	97.77	756
	OQ979238.1	Trichophyton mentagrophytes	Internal transcribed spacer 1, 5.8S ribosomal RNA, internal transcribed spacer 2, and large subunit ribosomal RNA	773	773	100%	97.77	864
Sample 6	MH791432.1	Trichophyton rubrum	18S ribosomal RNA, internal transcribe spacer 1, 5.8S Ribosomal RNA, internal transcribed spacer 2 and 28S Ribosomal RNA	1014	1014	100%	100	995
Sample 7	OR249962.1	Trichophyton rubrum	Internal transcribed spacer 1, 5.8S ribosomal RNA, internal transcribed spacer 2, and large subunit ribosomal RNA	977	977	100%	100	602
		Trichophyton violaceum	Small subunit ribosomal RNA, internal transcribed spacer 1, 5.8S ribosomal RNA, and internal transcribed spacer 2	977	977	100%	100	667
Sample 8	OR244359.1	Trichophyton indotineae	Small subunit ribosomal RNA, internal transcribed spacer 1, 5.8S ribosomal RNA, internal transcribed spacer 2, and large subunit ribosomal RNA	989	989	100%	100	676
	OQ978648.1	Trichophyton interdigitale	Internal transcribed spacer 1, 5.8S ribosomal RNA, internal transcribed spacer 2, and large subunit ribosomal RNA	989	989	100%	100	686
	OQ787386.1	Trichophyton mentagrophytes	Internal transcribed spacer 1, 5.8S ribosomal RNA, internal transcribed spacer 2, and large subunit ribosomal RNA	989	989	100%	100	648

The distribution of BLAST hits across different species is outlined in Table 2. The data categorize the number of hits per organism for each sample. For Samples 1, 2, 5, and 8, the majority of hits are attributed to *T. mentagrophytes*. However, these samples also contain a significant number of hits for *T. indotineae*, with fewer hits for* T. interdigitale*. On the other hand, Samples 3 and 6 are almost exclusively composed of hits for *T. rubru*m. Sample 4 primarily shows hits for *T. rubrum *but also includes a smaller proportion of hits for *Trichophyton soudanense*, along with a single hit for *Trichophyton kuryangei*. Lastly, Sample 7 is overwhelmingly specific to *T. rubrum*, comprising 99 hits, but also contains a single hit for *T. violaceum*.

**Table 2:** Summary of BLAST search for respective samples based on the number of hits and taxonomy BLAST: Basic Local Alignment Search Tool - Nucleotide

Query ID	Total Hits	List of Organism Hits	No. of Hits
Sample 1	105	Trichophyton indotineae	49
		Trichophyton interdigitale	11
		Trichophyton mentagrophytes	45
Sample 2	105	Trichophyton indotineae	43
		Trichophyton interdigitale	15
		Trichophyton mentagrophytes	47
Sample 3	100	Trichopython rubrum	100
Sample 4	101	Trichophyton rubrum	80
		Trichophyton soudanense	20
		Trichopython kuryangei	1
Sample 5	105	Trichopython indotineae	48
		Trichophyton interdigitale	12
		Trichophyton mentagrophytes	45
Sample 6	100	Trichophyton rubrum	100
Sample 7	100	Trichopython rubrum	99
		Trichopython violaceum	1
Sample 8	105	Trichophyton indotineae	43
		Trichophyton interdigitale	17
		Trichophyton mentagrophytes	45

## Discussion

Dermatophytosis is a very common yet neglected disease of the tropics. The present study was undertaken to understand the molecular character of eight representative *Trichophyton* sp., from repertoire stocks in the department of microbiology in a tertiary care hospital in the north eastern part of India. The present study relied on sequencing of the ITS region of 5.8SrDNA of dermatophytes. The aim of the study was to evaluate the accuracy of phenotypic identification and correlate it with molecular identification and to investigate the feasibility of using genotypic identification of dermatophytes on a routine basis.

The phenotypic identification of our isolates was confirmed in 87.1% (7/8)of our isolates by DNA sequencing using the ITS region. This is in phase with the studies of Li et al. [11] (81%, 102/126) and Diongue et al. (84.4%, 27/32) [12]. This variability could have arisen initially due to the subjectivity associated with conventional identification methods and due to the ambiguity inherent in the dermatophyte species complexes.

The present study has also highlighted the uncertainty associated with the usage of a pan-fungal primer such as the ITS in the speciation of dermatophytes. In the present study of the eight isolates sequenced, only two of the isolates exhibited 100% identity with that of *T. rubrum*. ITS was unable to differentiate the different species within the complexes, namely, *T. rubrum* complex and *T. mentagrophytes *complex. The BLASTn analysis exhibited significant hits for all species within the *T. mentagrophytes *complex, namely, *T. mentagrophytes* and *T. interdigitale*,and the novel species of *Trichophyton indotinae* similarly within the *T. rubrum *complex. Clear delineation of constituent species was not possible; in fact, species such as *T. soudanense* and *T. violaceum*, which are now described as independent species, were also included within it. These observations were in tandem with the observations of deHoog et al., Frais-de-Leon et al., and Normand et al. [13-15], but are against the studies of Nenoff et al. and Bhosale et al. [16,17].

The present study has also brought to the limelight the circulation of the novel dermatophyte species, namely, *T. indotinae* within the patients presenting at the our tertiary care hospital. This is the first of its kind study wherein the presence of this notorious dermatophyte has been brought to the notice of the medical fraternity in the north eastern part of India. *T. indotinae* has been reported to be isolated from many parts of the subcontinent in general and India in particular for the last few years. It has replaced almost all the previously reported species of dermatophytes and is the etiological agent implicated in epidemic-like upsurge of relapsing, recalcitrant, difficult-to-treat cases of dermatophytosis [18-25]. The most alarming is the high association of this dermatophyte with terbinafine resistance and an increasing minimum inhibitory concentration (MIC) of drugs such as griseofulvin, fluconazole, voriconazole, and a rapidly increasing MIC for itraconazole, thus limiting the treatment options available for all such patients [19-27].

The present study highlighted the comparison of phenotypic and molecular characteristics in a limited number of samples with the use of a specific molecular marker, which is one of the limitations of the present study. In the near future, it will be interesting to undertake studies with larger sample sizes and more molecular markers with higher discrimination power to counteract the intraspecies infidelities, thus negating any form of selection bias due to the arbitrary selection of isolates and limited sample size.

## Conclusions

The present study from the onset was conducted to understand the feasibility of using molecular analysis in the identification of dermatophytes on a day-to-day basis, especially in a resource-limited setting. Considering the ambiguity inherent to the morphological identification of dermatophytes, especially in the related taxa in our study isolates, the amplification reaction of ITS1-5.8S-ITS2 region did not provide any reliable alternative for identification of the *T. mentagrophytes* complex and *T. rubrum* complex, which had arisen out the intraspecies genetic homogeneity. Thus, molecular speciation based on ITS region flanking 5.8SrDNA was unable to clearly delineate whether a pan-fungal primer can ever actually replace the conventional mycological methods. Thus, more specific gene targets need to be analyzed so that molecular methods can come into routine use with respect to dermatophytes, failing which financial liability is bound to arise, especially in a resource-limited setting.

The present study was also able to elicit that *T. indotinae* responsible for epidemics of chronic dermatophytosis throughout India is also present in our population of the northeast, highlighting the importance of more specific phenotypic or genetic identifiers for such resistant isolates. It also beckons dermatologists in northeast India to be more vigilant and microbiological laboratories to build capacity for proper identification of all such resistant dermatophytes. The clinical implications of being complacent about this rapidly spreading dermatophyte could have devastating ramifications, especially in the northeastern part of India, as healthcare facilities in the region might not be equally equipped to deal with this silent epidemic.

## References

[1] Vos T, Abajobir AA, Abate KH (2017). Global, regional, and national incidence, prevalence, and years lived with disability for 328 diseases and injuries for 195 countries, 1990-2016: a systematic analysis for the Global Burden of Disease study 2016. Lancet.

[2] Havlickova B, Czaika VA, Friedrich M (2008). Epidemiological trends in skin mycoses worldwide. Mycoses.

[3] Ebert A, Monod M, Salamin K (2020). Alarming India-wide phenomenon of antifungal resistance in dermatophytes: a multicentre study. Mycoses.

[4] Lakshmanan A, Ganeshkumar P, Mohan SR, Hemamalini M, Madhavan R (2015). Epidemiological and clinical pattern of dermatomycoses in rural India. Indian J Med Microbiol.

[5] Nenoff P, Verma SB, Vasani R (2019). The current Indian epidemic of superficial dermatophytosis due to Trichophyton mentagrophytes-a molecular study. Mycoses.

[6] Hanumanthappa H, Sarojini K, Shilpashree P, Muddapur SB (2012). Clinicomycological study of 150 cases of dermatophytosis in a tertiary care hospital in South India. Indian J Dermatol.

[7] Gräser Y, Czaika V, Ohst T (2012). Diagnostic PCR of dermatophytes--an overview. J Dtsch Dermatol Ges.

[8] Hirai A, Kano R, Nakamura Y, Watanabe S, Hasegawa A (2003). Molecular taxonomy of dermatophytes and related fungi by chitin synthase 1 (CHS1) gene sequences. Antonie Van Leeuwenhoek.

[9] Kumar M, Mugunthan M (2018). Evaluation of three DNA extraction methods from fungal cultures. Med J Armed Forces India.

[10] Altschul SF, Gish W, Miller W, Myers EW, Lipman DJ (1990). Basic local alignment search tool. J Mol Biol.

[11] Li HC, Bouchara JP, Hsu MM, Barton R, Su S, Chang TC (2008). Identification of dermatophytes by sequence analysis of the rRNA gene internal transcribed spacer regions. J Med Microbiol.

[12] Diongue K, Bréchard L, Diallo MA (2019). A comparative study on phenotypic versus its-based molecular identification of dermatophytes isolated in Dakar, Senegal. Int J Microbiol.

[13] de Hoog GS, Dukik K, Monod M (2017). Toward a novel multilocus phylogenetic taxonomy for the dermatophytes. Mycopathologia.

[14] Frías-De-León MG, Martínez-Herrera E, Atoche-Diéguez CE, Cespón JL, Uribe B, Arenas R, Rodríguez-Cerdeira C (2020). Molecular identification of isolates of the Trichophyton mentagrophytes complex. Int J Med Sci.

[15] Normand AC, Packeu A, Cassagne C, Hendrickx M, Ranque S, Piarroux R (2018). Nucleotide sequence database comparison for routine dermatophyte identification by internal transcribed spacer 2 genetic region DNA barcoding. J Clin Microbiol.

[16] Nenoff P, Uhrlaß S, Verma SB, Panda S (2022). Trichophyton mentagrophytes ITS genotype VIII and Trichophyton indotineae: a terminological maze, or is it?. Indian J Dermatol Venereol Leprol.

[17] Bhosale NK, Prabha R, Munuswamy R, Pramodhini S, Easow JM (2022). A comparative study on the phenotypic versus molecular identification of clinical dermatophytes. J Pure Appl Microbiol.

[18] Chowdhary A, Singh A, Kaur A, Khurana A (2022). The emergence and worldwide spread of the species Trichophyton indotineae causing difficult-to-treat dermatophytosis: a new challenge in the management of dermatophytosis. PLoS Pathog.

[19] Singh A, Masih A, Khurana A (2018). High terbinafine resistance in Trichophyton interdigitale isolates in Delhi, India harbouring mutations in the squalene epoxidase gene. Mycoses.

[20] Singh A, Masih A, Monroy-Nieto J (2019). A unique multidrug-resistant clonal Trichophyton population distinct from Trichophyton mentagrophytes/Trichophyton interdigitale complex causing an ongoing alarming dermatophytosis outbreak in India: genomic insights and resistance profile. Fungal Genet Biol.

[21] Tang C, Kong X, Ahmed SA (2021). Taxonomy of the Trichophyton mentagrophytes/T. interdigitale species complex harboring the highly virulent, multiresistant genotype T. indotineae. Mycopathologia.

[22] Uhrlaß S, Verma SB, Gräser Y, Rezaei-Matehkolaei A, Hatami M, Schaller M, Nenoff P (2022). Trichophyton indotineae-an emerging pathogen causing recalcitrant dermatophytoses in India and worldwide-a multidimensional perspective. J Fungi (Basel).

[23] Verma SB, Panda S, Nenoff P (2021). The unprecedented epidemic-like scenario of dermatophytosis in India: II. diagnostic methods and taxonomical aspects. Indian J Dermatol Venereol Leprol.

[24] Nenoff P, Verma SB, Ebert A (2020). Spread of terbinafine-resistant Trichophyton mentagrophytes type VIII (India) in Germany-“the tip of the iceberg?”. J Fungi (Basel).

[25] Dogra S, Uprety S (2016). The menace of chronic and recurrent dermatophytosis in India: is the problem deeper than we perceive?. Indian Dermatol Online J.

[26] Rudramurthy SM, Shankarnarayan SA, Dogra S (2018). Mutation in the squalene epoxidase gene of Trichophyton interdigitale and Trichophyton rubrum associated with allylamine resistance. Antimicrob Agents Chemother.

[27] Yamada T, Maeda M, Alshahni MM (2017). Terbinafine resistance of Trichophyton clinical isolates caused by specific point mutations in the squalene epoxidase gene. Antimicrob Agents Chemother.

